# Marburg Virus Reverse Genetics Systems

**DOI:** 10.3390/v8060178

**Published:** 2016-06-22

**Authors:** Kristina Maria Schmidt, Elke Mühlberger

**Affiliations:** 1 Friedrich-Loeffler-Institut, Federal Research Institute for Animal Health, Institute of Novel and Emerging Infectious Diseases, Greifswald-Insel Riems 17493, Germany; kristina.schmidt@fli.bund.de; 2 Department of Microbiology, School of Medicine, Boston University, 620 Albany Street, Boston, MA 02118, USA; 3 National Emerging Infectious Diseases Laboratories (NEIDL), Boston University, 620 Albany Street, Boston, MA 02118, USA

**Keywords:** Marburg virus, Ebola virus, filoviruses, nonsegmented negative-sense RNA viruses, reverse genetics system, minigenome, full-length clones, virus-like particles, virus rescue, biosafety level 4

## Abstract

The highly pathogenic Marburg virus (MARV) is a member of the *Filoviridae* family and belongs to the group of nonsegmented negative-strand RNA viruses. Reverse genetics systems established for MARV have been used to study various aspects of the viral replication cycle, analyze host responses, image viral infection, and screen for antivirals. This article provides an overview of the currently established MARV reverse genetic systems based on minigenomes, infectious virus-like particles and full-length clones, and the research that has been conducted using these systems.

## 1. Introduction

### 1.1. Epidemiology

The *Filoviridae* family is subdivided into three distinct genera, *Marburgvirus*, *Ebolavirus* and *Cuevavirus*. The genus *Marburgvirus* includes a single species, *Marburg marburgvirus*, which is represented by two distinct viruses, Marburg virus (MARV) and Ravn virus (RAVV) [1]. Both MARV and RAVV cause a severe hemorrhagic disease in humans and susceptible animals [2]. The first reported MARV outbreak took place in Marburg and Frankfurt, Germany and Belgrade, Yugoslavia (now Serbia) in 1967, nine years prior to the first emergence of the better-known ebolaviruses, and was caused by infected African green monkeys imported from Uganda. Seven (22%) of the 31 infected patients succumbed to the disease (reviewed in [3]). All consecutive marburgvirus episodes and outbreaks were traced back to sub-Saharan Africa. The so far largest MARV outbreak took place in Uíge, Angola with 252 affected patients, many of them children, and a devastating case fatality rate of 90% [4,5]. This striking difference in survival rates between the 1967 MARV outbreak in resource-rich Europe, where the patients received aggressive medical treatment, and resource-poor Angola with suboptimal treatment options supports the observation during the most recent Ebola virus (EBOV) outbreak in West Africa that high-resource intensive care measurements significantly improve disease outcome [6]. However, other parameters, including transmission route and differences in the virulence of the viral strains might also have accounted for the observed differences in case fatality rates. 

Similar to ebolaviruses, human-to-human transmission of MARV mainly occurs via body fluids and requires close contact to infected patients or deceased. Sexual transmission was reported during the 1967 MARV outbreak (for review see [3]). Due to the severity of the disease and the lack of treatment and vaccination options, work with MARV and RAVV is restricted to biosafety level (BSL) 4 facilities. 

Filoviruses are zoonotic viruses. Strikingly, almost all primary marburgvirus infections in humans were traced back to caves inhabited by bats, and it was possible to isolate live MARV and RAVV viruses from the common Egyptian fruit bat (*Rousettus aegyptiacus* (*R.* *aegyptiacus*)) [7,8,9,10,11,12,13]. The marburgvirus bat isolates mirror the genetic diversity found in human isolates [10]. *R.* *aegyptiacus* bats experimentally infected with MARV did not show obvious signs of disease. Although viral titers determined from body fluids and tissues of the infected animals were generally moderate, oral and rectal shedding was observed in some, but not all, of the studies and could be a possible route of infection of humans [14,15,16]. However, MARV was not transmitted from experimentally infected to susceptible in-contact bats [17]. 

### 1.2. Virus Structure and Genome Oganization

#### 1.2.1. Viral Proteins

This article only provides a brief overview of the MARV proteins and their functions. For a more detailed description see [3,18].

The filamentous MARV particles consist of a host cell-derived membrane, seven viral proteins and the nonsegmented negative-sense RNA genome. The single surface protein, glycoprotein (GP), is inserted into the viral membrane [19]. GP is required for attachment, receptor binding and fusion, and enhances budding (reviewed in [20,21]). After synthesis in the endoplasmic reticulum (ER) and during its transport to the cell membrane, GP is cleaved in the *trans*-Golgi network in two subunits, GP_1_ and GP_2_ which are covalently linked [22]. While GP_1_ mediates attachment to the cell surface and receptor binding [20], membrane-bound GP_2_ contains the fusion domain [23]. In addition to its function in viral entry and budding, GP plays an important role as target protein for the development of antiviral therapeutics, including therapeutic monoclonal antibodies, and vaccines [24,25,26,27,28]. 

The viral protein (VP) 40 is a typical viral matrix protein and mediates budding [29,30,31]. In contrast to EBOV VP40 (eVP40), MARV VP40 (mVP40) antagonizes the Janus kinase/Signal Transducer and Activator of Transcription (JAK/STAT) signaling pathway and plays a crucial role as a virulence factor in host adaptation [32,33,34,35,36]. 

The helical MARV ribonucleoprotein complex, or nucleocapsid, is composed of five viral proteins, nucleoprotein (NP), VP35, VP30, large protein (L) and VP24, and the viral RNA. VP24, a viral protein unique to filoviruses, is loosely attached to the nucleocapsid [37,38]. VP24 is involved in viral particle release, possibly in nucleocapsid maturation, and might also play a role in the regulation of viral genome replication [39,40]. Recently, it has been shown that MARV VP24 (mVP24) competes with the nuclear transcription factor erythroid-derived 2 (Nrf2) for binding with Kelch-like ECH-associated protein 1 (Keap1), a negative regulator of Nrf2. This leads to the constitutive activation of the anti-oxidative stress response, including the expression of cytoprotective genes, in MARV-infected cells [41,42]. Intriguingly, EBOV VP24 (eVP24) does not bind to Keap1. 

Both the MARV genomic and antigenomic RNAs are encapsidated by NP. NP is the driving force for nucleocapsid formation. It self-assembles along the viral RNA and interacts with the other nucleocapsid proteins either directly or via protein linkers to form the nucleocapsids [37,38,43,44]. MARV NP (mNP) is also involved in the recruitment of cellular components of the endosomal sorting complexes required for transport (ESCRT) to support intracellular transport and budding of viral particles [45,46,47].

VP35 is a multifunctional protein involved in nucleocapsid formation, viral RNA synthesis, and the suppression of antiviral responses. It interacts with NP and seems to play an important role in chaperoning the formation of the NP-RNA complex. Recent crystal structure analyses revealed that EBOV VP35 (eVP35) contains an intrinsically disordered region that prevents EBOV NP (eNP) oligomerization and releases RNA from eNP-RNA complexes [48,49]. Although comparable structural analyses are not yet available for MARV VP35 (mVP35)-NP-RNA complexes, it is conceivable that similar encapsidation strategies may apply. Besides its function as a structural nucleocapsid component, VP35 is a polymerase cofactor and, together with L, forms the viral RNA-dependent RNA polymerase complex. VP35 binds to both NP and L and connects L to the NP-RNA complex [38,50]. L is the enzymatic subunit of the polymerase complex [3,18,51]. VP35 is not only required for viral replication and transcription, it also interferes with the host innate immune response by blocking retinoic acid-inducible gene 1 (RIG-I)-like receptor-mediated type I interferon (IFN) induction. mVP35 can bind to double-strand RNA (dsRNA), and the integrity of its dsRNA binding domain is essential for its antagonistic properties [52,53,54,55,56]. 

The function of the nucleoprotein VP30 in the MARV replication cycle is poorly understood. Although it is tightly associated with the nucleocapsid complex, it is dispensable for proper nucleocapsid formation [37,38,57]. In contrast to EBOV VP30 (eVP30), which is a transcription activator and significantly enhances transcriptional activity in an EBOV minigenome system [58,59], MARV VP30 (mVP30) is not essential for efficient transcription of MARV minigenomes and has only moderate effects on minigenome-driven reporter gene expression [40,60,61]. However, rescue of full-length MARV clones was only successful in the presence of VP30 indicating an important role of mVP30 during the MARV replication cycle [62]. This observation was supported by a small interfering RNA (siRNA) study, in which downregulation of VP30 in MARV-infected cells strongly reduced viral protein amounts [63]. Despite the obvious functional differences observed in the minigenome systems, mVP30 and eVP30 are structurally closely related. This close relationship is reflected by functional similarities. Thus, mVP30 was able to enhance transcription in an EBOV minigenome system, albeit with reduced efficiency [58]. Vice versa, rescue of MARV full-length clones was also successful when mVP30 was replaced by eVP30 [62]. In summary, mVP30 is an essential component of the MARV replication cycle, but its function is less well studied than that of its EBOV counterpart. 

#### 1.2.2. Genome Organization

**Figure 1:**
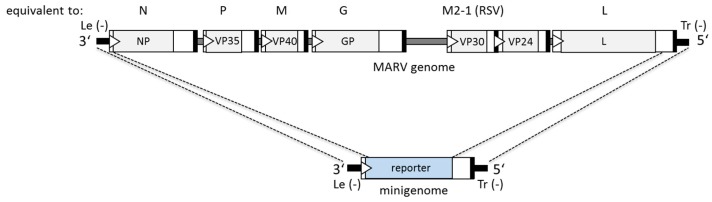
Scheme of the Marburg virus (MARV) genome (**top**) and the minigenome (**bottom**) in negative-sense orientation. Coding regions of the seven MARV genes are shown as grey boxes in the genome. The homologous proteins of other nonsegmented negative-sense (NNS) RNA viruses are indicated above the genome. In the minigenome, the viral genes are replaced by a single reporter gene, which is flanked by the MARV leader and trailer regions, the nontranslated region of the *nucleoprotein* (*NP*) gene containing the gene start (GS) signal, and the nontranslated region of the *large protein* (*L*) gene containing the gene end (GE) signal. Black lines, leader and trailer regions; gray lines, intergenic regions; white bars, nontranslated regions; white arrow heads, GS signals; black bars, GE signals; blue box, reporter gene.

The nonsegmented negative-sense RNA genome of MARV is about 19 kb in length and contains seven monocistronic genes encoding the seven viral proteins (Figure 1, top). Each gene is flanked by conserved gene start (GS) and gene end (GE) signals which are recognized by the viral polymerase as the sites of transcription initiation and termination. The genes are either separated by intergenic regions of variable length or they overlap [3,51,64,65]. Regulatory cis-acting elements containing the promoter regions are located at the 3′ and 5′ ends of the genome (Figure 1, top). These regions are the leader (3′ end of the negative-sense genome) and the trailer (5′ end of the genome). The leader of the MARV Musoke isolate is 48 nucleotides (nts) in length and contains the first promoter element of the bipartite replication promoter. The second promoter element is located within the 3′ untranslated region (negative-sense orientation) of the NP gene [66]. The transcription promoter is also located in the leader but is not mapped yet. The trailer region contains the complementary replication promoter which is used to produce genomes from the positive-sense antigenomic RNA template. 

MARV transcription follows the stop-start model postulated for all nonsegmented negative-sense (NNS) RNA viruses [67]. The viral polymerase enters the genome at a single transcription promoter located within the leader and scans the genome until it reaches the GS signal of the first gene where it initiates transcription. The nascent mRNA is capped by the polymerase complex immediately after transcription initiation. The polymerase moves along the template until it recognizes a GE signal, leading to transcription termination. Using an unusual stuttering mechanism, the polymerase adds a poly-A tail to the nascent mRNA strand. It then scans for the next GS signal to initiate transcription of the following gene. If the polymerase complex falls off the template, it has to re-enter the genome at the transcription promoter located in the leader. This sequential transcription leads to an mRNA gradient with the 3′ proximal genes being more frequently transcribed than the 5′ proximal genes [65]. 

**Figure 2:**
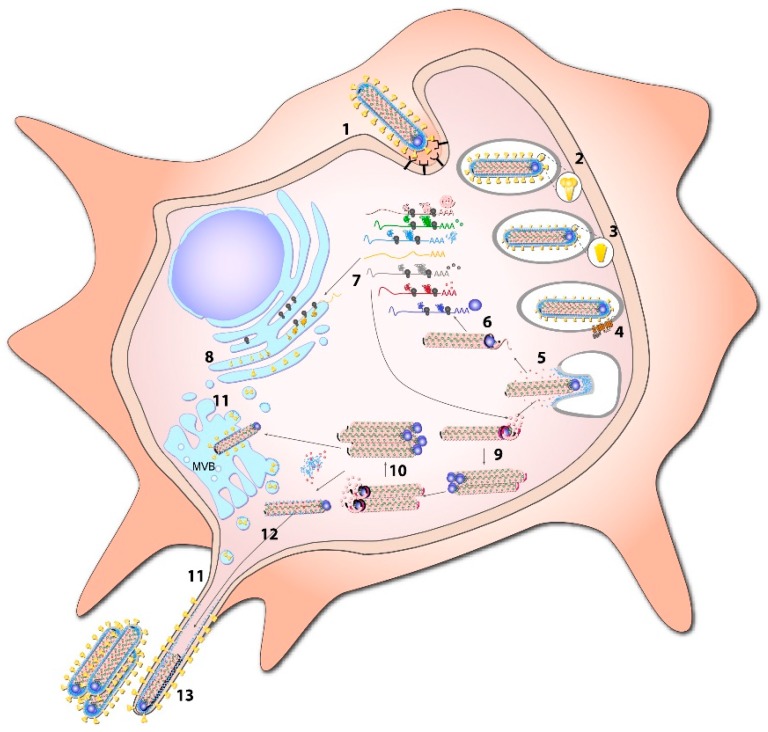
MARV replication cycle. MARV attaches to the surface of target cells by binding to attachment factors (**1**); following endocytosis (**2**); glycoprotein subunit GP_1_ is cleaved by endosomal proteases (**3**) facilitating binding to Niemann-Pick C1 (NPC1); the entry receptor (**4**); fusion is mediated in a pH-dependent manner by glycoprotein subunit GP_2_. Following release of viral nucleocapsid into the cytosol (**5**); transcription of the viral genome takes place (**6**); mRNA is subsequently translated by the host cell machinery (**7**); synthesis of GP takes place at the endoplasmic reticulum (ER) and undergoes multiple post-translational modifications on its way through the classical secretory pathway (**8**); positive sense antigenomes are synthesized from the incoming viral genomes (**9**); these intermediate products then serve as templates to replicate new negative-sense genomes (**10**); after cleavage in the Golgi, GP is transported to multivesicular bodies (MVB) and to the cell membrane where budding takes place preferentially from filopodia (**11**); nucleocapsids and viral protein (VP) 24 are also recruited to sites of viral budding (**12**); which is mainly driven by VP40 (**13**); figure and modified legend from [3].

**Figure 3:**
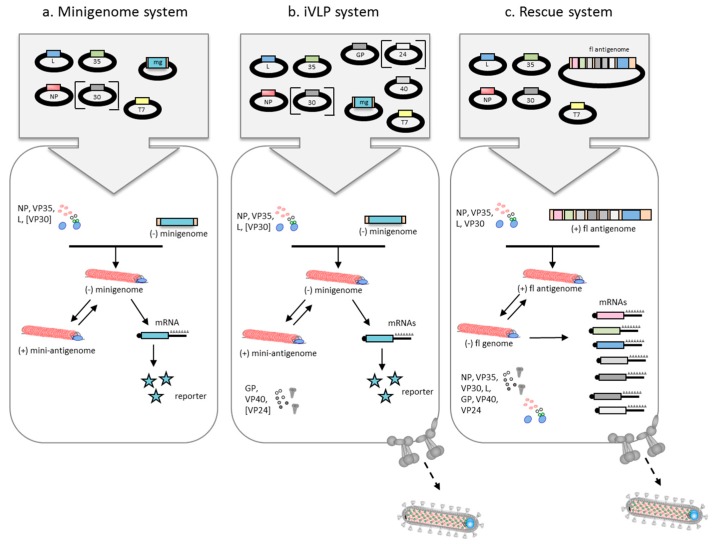
MARV reverse genetic systems. (**a**) Minigenome system; (**b**) infectious virus-like particles (iVLP) system; and (**c**) rescue system. In all systems, cells are transfected with the expression plasmids for the nucleocapsid proteins (NP, VP35, VP30, large protein (L)). For the iVLP system, the expression plasmids for GP, VP40 and VP24 are also included to facilitate budding. Viral proteins, which are not essential for the respective system, are bracketed. The T7 RNA polymerase (T7), which is required for the expression of the minigenome (mg), the full-length antigenome (fl antigenome) and, depending on the plasmid backbone, for the expression of the support proteins, can be provided by using T7-expressing cells, infection with T7-expressing viruses or by transfecting a T7 expression plasmid. (**a**,**b**) Minigenomic or (**c**) antigenomic cDNA is transcribed by T7 resulting in negative-sense minigenomic RNA or positive-sense fl antigenomic RNA. Encapsidation is mediated by the expressed viral nucleocapsid proteins. (**a**,**b**) The minigenome is replicated via a positive-sense mini-antigenome intermediate. Transcription of the minigenome leads to reporter gene expression; (**c**) The fl antigenome serves as the template for the production of viral genomic RNA which in turn is the template for viral mRNA as well as antigenome production. (**b**,**c**) In the iVLP and rescue systems, viral particles are released from the transfected cells. The iVLPs contain minigenome RNA, while the rescue system leads to the production of infectious MARV particles.

During genome replication, the viral polymerase binds to the bipartite replication promoter and generates a full-length complementary copy of the genome, the antigenome. The GS and GE signals are ignored when the polymerase is in replication mode. The antigenome in turn serves as the template for the production of viral genomes (Figure 2 and Figure 3). Both genome and antigenome are encapsidated by the nucleocapsid proteins. This is not the case for the viral mRNAs [3,18,51,60].

### 1.3. Replication Cycle

A detailed description of the MARV replication cycle is provided in [3]. In brief, uptake of MARV particles is mediated by GP (Figure 2). Following initial GP-mediated attachment to various cell surface proteins, the viral particles are endocytosed (reviewed in [20,68]). It is likely that macropinocytosis plays a crucial role in MARV entry as this mechanism was identified as a major uptake pathway for EBOV particles [69,70]. Endosomal cleavage of GP_1_ by host cell proteases is required for binding to its receptor, the endosomal protein Niemann-Pick C1 (NPC1) [71,72,73,74]. Fusion with the cellular membrane is preceded by a pH-dependent structural rearrangement of the GP_2_ subunit which contains the fusion peptide [23,75]. After fusion of the viral and cellular membranes, the nucleocapsids are released into the cytoplasm where transcription and replication of the viral genomes take place (Figure 2). The viral monocistronic mRNAs are translated by cellular machinery to produce viral proteins. The newly synthesized viral genomes (and antigenomes) are encapsidated by the nucleocapsid proteins and assemble to large, highly ordered structures in the cytoplasm of the infected cells, the so-called inclusions [43,76,77]. These inclusions are believed to be the sites of viral genome replication and nucleocapsid maturation [37]. Mature nucleocapsids are transported along actin filaments from the inclusions to the sites of budding by exploiting components of the ESCRT complex, including tumor susceptibility gene 101 (Tsg101) [45,46,47]. Budding of viral particles is mainly mediated by VP40 and occurs internally at multivesicular bodies and at the plasma membrane preferentially from filopodia [29,77,78,79,80]. Viral particle release is enhanced by NP, GP and VP24 [39,45,46,81]. Studies on MARV budding in different cell models suggest that cell-type specific components determine whether the viral particles are released from the apical or the basolateral membrane [82,83,84].

## 2. MARV Reverse Genetics Systems 

Per definition, reverse genetics is the functional analysis of genes by examining the phenotypic effects of targeted gene alterations. Reverse genetics systems have been successfully developed for numerous NNS RNA viruses of the order *Mononegavirales* [85,86]. This powerful technology has been used to address questions regarding all aspects of the viral infection, including viral genome replication, pathogenesis, and virus-host interactions. In addition, reverse genetics systems have been instrumental for the development of vaccines and antiviral screening assays (reviewed in [86]). In contrast to positive-sense RNA viruses, whose genome is used as an mRNA and is sufficient for virus particle formation when transfected into cells, the minimal infectious unit for NNS RNA viruses is the ribonucleoprotein complex, in which the viral RNA is encapsidated by the viral ribonucleoproteins before it can serve as a functional template to initiate viral transcription and genome replication (reviewed in [85,86,87]). Because neither the genome nor the antigenome of NNS RNA viruses can be used as an mRNA to generate viral proteins, the viral proteins required for viral transcription and genome replication have to be provided in *trans*.

Reverse genetics approaches for NNS RNA viruses range from minigenome systems to replicons, infectious virus-like particles (iVLPs) and full-length rescue systems. Since targeted genetic manipulation of viral RNA genomes is not possible yet, a tremendous advantage of all these systems is that the reverse transcribed cDNA copy of the RNA genome allows for the insertion of mutations and additional transcription units (ATUs; e.g., reporter genes). The first reverse genetics system established for filoviruses was the MARV minigenome system [60]. The different established MARV reverse genetics systems are summarized in Table 1 and described in the following overview.

### 2.1. Minigenome Systems

Minigenomes are truncated versions of the viral genome as described below. The first cDNA-based minigenome system for NNS RNA viruses was generated for vesicular stomatitis virus (VSV) in the Ball and Wertz laboratories in 1992 [98]. The development of an exclusively cDNA-based minigenome system for NNS RNA viruses was hampered for a long time by the requirement of precise minigenome ends to initiate replication and transcription by the viral polymerase complex. To overcome this issue, Pattnaik and colleagues came up with an elegantly designed plasmid containing a VSV minigenome. In this plasmid, the minigenome was inserted immediately downstream of the T7 RNA polymerase promoter leading to the generation of discrete 5′ ends (negative-sense orientation) when the minigenome was transcribed by the T7 RNA polymerase in transfected cells. To generate precise 3′ ends, the hepatitis delta virus (HDV) ribozyme sequence was inserted downstream of the minigenome sequence. Following T7 RNA polymerase-mediated transcription of the minigenome-ribozyme hybrid RNA, the ribozyme sequence was removed through its autocatalytic cleavage activity thereby generating exact 3′ minigenome ends [98,99,100]. This elegant approach led to a tremendous boost in generating reverse genetics systems for NNS RNA viruses including MARV [85]. 

The first MARV minigenome system was generated in the Mühlberger laboratory based on A. Ball’s vector p 2.0 in which the MARV minigenome sequence was inserted between the T7 RNA polymerase promoter and the HDV ribozyme [60,98]. To our knowledge, all subsequently generated MARV minigenomes follow a similar design [61]. The minimal requirement for replicating minigenomes is the possession of the replication promoters at both minigenome ends [60]. Transcribing minigenomes also need the transcription promoter located at the 3′ end of the minigenome (negative-sense orientation) and virus-specific GS and GE signals flanking each gene [60,66]. The viral genes are removed and replaced by one (monocistronic minigenomes) or more (polycistronic minigenomes) non-viral genes, usually reporter genes. Reporter gene expression is an easy readout for successful minigenome transcription and replication. 

In the first generation of monocistronic MARV minigenomes, the reporter gene was flanked by 106 nts of the 3′ end of the MARV genome and 439 nts of the 5′ end of the genome. The 3′ end of the minigenome preceding the reporter gene spans the leader, the NP GS signal and the 3′ non-translated region of the NP gene (negative-sense orientation). The bipartite replication promoter of MARV is contained within this sequence [66]. The 5′ end of the minigenome downstream of the reporter gene consists of the 5′ non-translated region of the L gene, the L GE signal and the trailer region of the viral genome. Reporter gene transcription is driven by the GS signal of NP for initiation and the GE signal of L for termination [60] (Figure 1). 

Variations of this minigenome design include replacing the leader region with a copy-back trailer region, inserting the minigenome in positive-sense orientation [60], generating chimeric MARV/EBOV minigenomes [66] and extending the monocistronic minigenome to bicistronic MARV minigenomes by inserting two reporter genes separated by a MARV-specific intergenic region [90]. In addition, minigenomes with different reporter genes have been generated, including chloramphenicol acetyltransferase (CAT), firefly luciferase (fLuc; Mühlberger, unpublished), Renilla luciferase (rLuc), Gaussia luciferase (gLuc), green fluorescent protein (GFP) and Cypridina luciferase (cLuc) [40,60,61,90,91]. The rLuc and gLuc reporter minigenome systems have the potential to be used as high-throughput antiviral screening platforms [40,91]. 

All recombinant MARV replication and transcription systems follow the same basic approach. The MARV minigenome plasmid is transfected along with the plasmids encoding the nucleocapsid proteins NP, VP35, and L which are the minimal requirement for MARV minigenome replication and transcription [60] (Figure 3a). In contrast to the Ebola virus minigenome assay [58], the nucleocapsid protein VP30 is not required for efficient transcription in the MARV minigenome system [60], although addition of VP30 moderately enhanced minigenome activity [40] and led to an increase of GFP-positive cells in a GFP-based MARV minigenome system [61]. However, VP30 is essential for the rescue of MARV from full-length cDNA clones (see Section 2.3). Initial transcription of the minigenome by the T7 RNA polymerase takes place in the cytoplasm. There are different approaches to provide the T7 RNA polymerase in *trans,* including (i) expression from a plasmid encoding the T7 RNA polymerase under the control of a eukaryotic promoter (e.g., pCAGGS-T7 [101]); (ii) use of a cell line constitutively expressing the T7 RNA polymerase (e.g., the baby hamster kidney cell line BSR-T5/7 [102]); or (iii) infection with a recombinant vaccinia virus encoding the T7 RNA polymerase (MVA-T7) [103]. 

The T7 RNA polymerase-derived minigenome RNA is then used as a template for transcription and replication by the newly synthesized nucleocapsid proteins (Figure 3a). There are different ways to express the support proteins: the nucleocapsid proteins genes are either transcribed by the T7 RNA polymerase in the cytoplasm [60] or by the cellular RNA polymerase II in the nucleus of the transfected cells [40,61]. It is conceivable that the MARV mRNAs contain cryptic splice sites which can be targeted by the spliceosome when transcription takes place in the nucleus, leading to unwanted splicing events and consequently, reduced protein expression. This issue can be overcome using codon-optimized constructs in which putative cryptic splice sites are eliminated [104]. Using codon-optimized support plasmids significantly increased reporter gene expression in a MARV minigenome system and was instrumental for the successful recovery of full-length MARV clones [61,91].

Minigenome systems are particularly well suited to dissect *cis*-acting genetic elements, including replication and transcription promoters, transcription start and stop signals, intergenic regions and RNA editing signals. The MARV minigenome has been used to analyze *cis*-acting signals involved in viral transcription and replication [58,60], to map the replication promoter and to characterize the genus-specificity of *cis*-acting signals in chimeric MARV/EBOV minigenomes [66]. Further, chimeric minigenomes were used to compare the impact of the intergenic regions of the MARV isolates Musoke and Angola on transcriptional activity [90]. 

In addition to *cis*-acting functions, MARV minigenome systems have been used to analyze *trans*-acting factors relevant for minigenome transcription and replication. This includes determining the protein requirement for replication and transcription and optimizing the amount and ratio of the support plasmids [40,60,61]. The optimal protein amounts may vary and have to be adapted to each system, especially the ratio of NP to VP35, which has been found to be very critical [60]. Functional studies of the nucleocapsid proteins have been instrumental to map NP phosphorylation sites critical for minigenome activity and protein binding domains on VP35 involved in the formation of a functional nucleocapsid complex [50,88,89,92]. 

### 2.2. Infectious VLP Systems

To overcome the limitations that minigenome systems are restricted to replication and transcription, other systems to model multiple steps of the viral life cycle have been developed. Expression of VP40 in the absence of other viral proteins leads to the formation and release of VLPs that are structurally similar to MARV particles and can incorporate GP [105,106]. The iVLP system combines the VLP approach with the minigenome system. 

The first iVLP system for MARV was published by the Becker laboratory in 2010 [40]. In this system, multiple steps of the MARV replication cycle including replication/transcription, morphogenesis, budding, and infection of target cells can be examined (Figure 3b). In contrast to VLPs, the iVLPs contain nucleocapsids that are able to mediate replication and transcription (Figure 3b). To generate iVLPs, cells are transfected with the minigenome plasmid along with expression plasmids for all viral proteins and the T7 RNA polymerase. Expression of the viral proteins leads to the formation of iVLPs containing nucleocapsids. The iVLPs are released from the cell by budding and can be used to infect naive target cells. A second replication cycle can only be mediated by this system if the target cells express the viral nucleocapsid proteins [40,92,94]. Similar to the minigenome system, NP, VP35 and L, but not VP30, were essential for iVLP formation. iVLP formation was further strictly dependent on the expression of VP40 and was increased in the presence of GP, whereas VP24 did not affect iVLP formation [40]. Titration experiments to optimize the system revealed that higher amounts of VP40 and VP24 had a negative impact on viral replication and/or transcription. While the release mechanism of iVLPs appeared to be similar to that of MARV particles from infected cells, the morphology and length of these iVLPs and their nucleocapsid cores varied, which influenced their infectivity [40]. Chimeric iVLPs composed of different combinations of EBOV and MARV components revealed a strict genus-specific interaction of VP40 with nucleocapsids, while VP40 tolerated GP from a different filovirus genus [94]. Further functional studies using the iVLP system include the characterization of viral proteins, protein domains and post-translational modifications crucial for various steps of the viral replication cycle [30,92,93]. This system has also been used to analyze the role of VP40 in host adaptation [36] and to determine neutralization titers of MARV-specific antisera [40]. 

### 2.3. Rescue Systems

The first rescue of NNS RNA viruses entirely from cDNA was achieved for rabies virus by the Conzelmann laboratory in 1994 [107]. Their approach was based on generating full-length positive-sense antigenomes from plasmid DNA instead of negative-sense genome transcripts. Rescue of recombinant NNS RNA viruses from cDNA requires de novo formation of replication-competent nucleocapsids using the T7 RNA polymerase derived-genomic (or antigenomic) RNA and the nucleocapsid proteins. In unsuccessful approaches that used the negative-sense RNA genome, the mRNAs of the support proteins (N, P, and L) might hybridize to the primary transcript of the T7 polymerase-derived genomic RNA, preventing the assembly of the genome into nucleocapsid complexes. The new approach, starting with the full-length positive-strand transcript, overcame this issue because the antigenome and the nucleocapsid protein mRNAs are in the same orientation and cannot hybridize. Despite ongoing efforts to develop rescue systems based on negative-sense full-length viral RNA, only few laboratories were successful using this approach. In addition, the established negative-sense RNA rescue systems were much less efficient compared to the respective positive-sense RNA approach [108,109]. As of date, rescue systems have been established for multiple members of the *Rhabdo*-, *Paramyxo*-, *Borna*- and *Filoviridae* families, and the list is still growing (reviewed in [85,86]). 

The first MARV rescue system was published by the Mühlberger laboratory in 2006 using the positive-sense approach [62]. The cDNA encoding the MARV Musoke antigenomic RNA was inserted into an expression vector under the control of the T7 RNA polymerase promoter followed by the HDV ribozyme and a T7 RNA polymerase termination motif. Concomitantly with synthesis by the T7 RNA polymerase, the MARV antigenome is encapsidated by the viral support proteins, forming the nucleocapsid which is used as a template for the synthesis of the negative-sense genomic RNA (Figure 3c). Nucleocapsid complexes containing the genomic RNA are the templates for synthesis of the viral mRNAs and the antigenomic RNA. Since all viral proteins are expressed from the viral genome, all steps of the viral replication cycle, including particle formation and budding, are mediated. Successful rescue was achieved by transfecting T7 RNA polymerase-expressing cells with plasmids encoding NP, VP35, L, and VP30 along with the full-length antigenome plasmid (Figure 3c). In contrast to the minigenome and iVLP systems, VP30 was essential for virus rescue [62]. To our knowledge, recombinant MARV (rMARV) systems have been established in three laboratories [61,62,95]. In contrast to the minigenome and iVLP systems, the rescue system can only be used in a BSL-4 setting because infectious MARV is generated. Rescue of rMARV can be challenging and was significantly improved by using codon-optimized support plasmids for the expression of the viral support proteins [61,91]. Attempts to rescue MARV from negative-sense genomic RNA were not successful [61].

Recombinant NNS RNA viruses, including MARV, are useful tools to study multiple aspects of the viral replication cycle, host-virus interactions, immune responses, and host adaptation mechanisms. Furthermore, they have been used in translational approaches for vaccine development and oncolytic therapy [110]. There are two main approaches of how recombinant NNS RNA viruses can be modified: (i) targeted mutagenesis of viral genes for functional analyses or to generate attenuated vaccine candidates; (ii) insertion of ATUs to image infection or to generate vectors for the expression of foreign genes (e.g., vaccine candidates, oncolytic viruses) [110,111]. rMARV clones have been generated by both approaches. 

#### 2.3.1. Targeted Mutagenesis of rMARV

The MARV full-length system was used to verify the function of *cis*-acting elements located within the viral replication promoter, which had originally been identified using the minigenome system [66]. It was also used to analyze the function of viral proteins involved in replication and transcription, including VP30 and L [61,62]. Functional studies on viral entry and particle release revealed that truncations of the cytoplasmic domain of GP led to growth defects and impaired entry [96]. Mutational analysis of a late domain motif within MARV NP, which is involved in the recruitment of the ESCRT protein Tsg101 [45], has provided insights into the role of NP in the transport of nucleocapsids to the sites of budding [46]. The filovirus rescue systems have also been used to study genus-specificity of the support plasmids. MARV was rescued using eVP30 as a support plasmid [62], and EBOV was successfully rescued using all four MARV support proteins (NP, VP35, VP30, L) [97]. Intriguingly, EBOV minigenomes were not replicated and transcribed using MARV support proteins [58], illustrating the sensitivity of the full-length rescue system that leads to autonomous viral replication and transcription after the jump-start transfection, using the viral proteins produced from the viral genomes. Targeted mutagenesis of viral proteins was further used to study the impact of adaptive mutations in the VP40 gene of guinea pig-adapted MARV and revealed increased fitness and higher infectivity in guinea pig cells infected with rMARV containing the adaptive mutations in the VP40 gene [36]. A bat-derived rMARV carrying mutations in the IFN inhibitory domain of VP35 was used to confirm the immunosuppressive functions of VP35 in the context of viral infection [54].

#### 2.3.2. rMARV Containing ATUs

Insertion of a gene encoding a fluorescent protein into the MARV genome has been used as a strategy to visualize viral infection. The first MARV construct (Musoke isolate) expressing a foreign gene contained the GFP gene as an independent transcription unit inserted between the VP35 and VP40 genes. To do this, the intergenic region between the VP35 and VP40 genes was mutated to an *Avr*II restriction site which was used to insert the GFP gene flanked by MARV-specific GS and GE sequences. The rescued virus showed slightly reduced replication kinetics in Vero cells compared to the MARV Musoke wild type. This might be attributed to a decrease in downstream gene expression due to the insertion of an ATU [77]. rMARV-GFP was used to detect virus-infected cells using live-cell imaging. It was observed that viral spread occurred mainly through cell-to-cell contact and from cell protrusions, and was promoted by viral replication in actively dividing cells. Intriguingly, GFP accumulated in MARV-derived inclusions in the infected cells [77]. The same strategy was used to insert a second copy of the MARV VP40 gene fused to the red fluorescent protein (RFP) gene into the *Avr*II restriction site, leading to the expression of a fluoresecentVP40 fusion protein in addition to unlabeled VP40 [80]. This virus was used for high resolution live-cell imaging studies to analyze the transport of MARV nucleocapsids to the sites of budding. Imaging of single nucleocapsid particles was achieved by providing VP30 fused to GFP in *trans*. This dual-color live-cell imaging approach allowed for simultaneous visualization of the nucleocapsids and VP40 in infected cells. It was shown that the nucleocapsids are transported along actin filaments to the plasma membrane where they associate with VP40 to be released from filopodia [80]. 

ATUs have also been inserted in rMARV clones based on the 371Bat isolate. 371Bat rMARV, in which the GFP gene was inserted between the NP and VP35 genes, grew to similar titers as the wild type virus in Vero cells, but was attenuated in primary human macrophages [61]. Similar to the GFP-containing MARV Musoke clone [77], the expression levels of viral genes located downstream of the ATU were decreased in cells infected with 371Bat rMARV-GFP [61]. Compared to wild type virus, infection with 371Bat rMARV-GFP elicited an increased inflammatory response in primary human macrophages. As a possible explanation for this observation, Albarino and colleagues suggested that the ability of the GFP-containing virus to counteract antiviral responses might be perturbed by the decreased expression levels of viral immune suppressors, such as VP35 and VP40 [61]. Bat-derived rMARV expressing gLuc from an ATU inserted between the NP and VP35 genes was generated to establish a high-throughput screening platform to test antivirals [91].

## 3. Conclusions

The various MARV reverse genetics systems have considerably contributed to our current understanding of the MARV replication cycle, MARV-host interactions, host specificity, pathogenesis, and antiviral treatment options. While the rescue systems result in the production of genetically engineered virus which can be used for all kinds of infection studies, including live-cell imaging approaches, they are restricted to a BSL-4 setting. Model systems that can be used to study various aspects of the MARV replication cycle under BSL-2 conditions because they do not lead to the production of infectious MARV include minigenome and iVLP systems. Although these model systems have certain restrictions compared to the rescue system, they are highly beneficial due to the lower biosafety level requirements. Despite the threat that filovirus disease poses to global public health, MARV, and to an even larger extent RAVV, are severely under-investigated, and there are many gaps in our understanding of MARV and RAVV infections. It is, therefore, desirable that the availability of MARV reverse genetics systems will stimulate more research activity on these important emerging pathogens.

## Figures and Tables

**Table 1:** List of Marburg virus (MARV) reverse genetics systems.

System	Isolate	Used For	Publications
minigenome	Musoke	Defining the role of viral proteins in transcription and replication, genus-specificity of viral proteins in replication/transcription, amount and ratio of nucleocapsid proteins, functional studies of nucleocapsid proteins, analyzing *cis*-acting elements and their genus-specificity	[50,58,60,66,88,89,90]
minigenome	371Bat	Optimization of system with codon-optimized support plasmids, establishing high throughput antiviral screening assay, analyzing immune modulatory functions of viral proteins	[54,61,91]
iVLP	Musoke	Release and infectivity of iVLPs, titration of support plasmids for iVLP assay, genus-specificity of viral proteins in budding, testing of neutralizing antibodies, defining the role of viral proteins in the viral replication cycle, host adaptation	[30,36,40,92,93,94]
rescue system	Musoke	Role of VP30 for virus replication, analyzing *cis*-acting elements, live-cell imaging, transport and release of nucleocapsids, assembly of viral envelope, host adaptation	[36,46,62,66,77,80,95,96]
rescue system	371Bat	Host response and immune modulatory activity, establishing high throughput antiviral screening assay	[54,61,91]
rescue system	Ebola virus Mayinga	Rescue of Ebola virus with MARV Musoke support plasmids	[97]

## References

[1] Bukreyev A.A., Chandran K., Dolnik O., Dye J.M., Ebihara H., Leroy E.M., Muhlberger E., Netesov S.V., Patterson J.L., Paweska J.T. (2014). Discussions and decisions of the 2012–2014 international committee on taxonomy of viruses (ICTV) *filoviridae* study group, January 2012–June 2013. Arch. Virol..

[2] Cross R.W., Fenton K.A., Geisbert J.B., Ebihara H., Mire C.E., Geisbert T.W. (2015). Comparison of the pathogenesis of the Angola and RAVN strains of Marburg virus in the outbred guinea pig model. J. Infect. Dis..

[3] Brauburger K., Hume A.J., Mühlberger E., Olejnik J. (2012). Forty-five years of Marburg virus research. Viruses.

[4] Towner J.S., Khristova M.L., Sealy T.K., Vincent M.J., Erickson B.R., Bawiec D.A., Hartman A.L., Comer J.A., Zaki S.R., Ströher U. (2006). *Marburgvirus* genomics and association with a large hemorrhagic fever outbreak in Angola. J. Virol..

[5] Ligon B.L. (2005). Outbreak of Marburg hemorrhagic fever in Angola: A review of the history of the disease and its biological aspects. Semin. Pediatr. Infect. Dis..

[6] Uyeki T.M., Mehta A.K., Davey R.T., Liddell A.M., Wolf T., Vetter P., Schmiedel S., Grunewald T., Jacobs M., Arribas J.R. (2016). Clinical management of Ebola virus disease in the United States and Europe. N. Engl. J. Med..

[7] Towner J.S., Pourrut X., Albarino C.G., Nkogue C.N., Bird B.H., Grard G., Ksiazek T.G., Gonzalez J.P., Nichol S.T., Leroy E.M. (2007). Marburg virus infection detected in a common African bat. PLoS ONE.

[8] Swanepoel R., Smit S.B., Rollin P.E., Formenty P., Leman P.A., Kemp A., Burt F.J., Grobbelaar A.A., Croft J., Bausch D.G. (2007). Studies of reservoir hosts for Marburg virus. Emerg. Infect. Dis..

[9] Pourrut X., Souris M., Towner J.S., Rollin P.E., Nichol S.T., Gonzalez J.P., Leroy E. (2009). Large serological survey showing cocirculation of Ebola and Marburg viruses in Gabonese bat populations, and a high seroprevalence of both viruses in *Rousettus aegyptiacus*. BMC Infect. Dis..

[10] Towner J.S., Amman B.R., Sealy T.K., Carroll S.A., Comer J.A., Kemp A., Swanepoel R., Paddock C.D., Balinandi S., Khristova M.L. (2009). Isolation of genetically diverse Marburg viruses from Egyptian fruit bats. PLoS Pathog..

[11] Adjemian J., Farnon E.C., Tschioko F., Wamala J.F., Byaruhanga E., Bwire G.S., Kansiime E., Kagirita A., Ahimbisibwe S., Katunguka F. (2011). Outbreak of Marburg hemorrhagic fever among miners in Kamwenge and Ibanda Districts, Uganda, 2007. J. Infect. Dis..

[12] Amman B.R., Carroll S.A., Reed Z.D., Sealy T.K., Balinandi S., Swanepoel R., Kemp A., Erickson B.R., Comer J.A., Campbell S. (2012). Seasonal pulses of Marburg virus circulation in juvenile *Rousettus aegyptiacus* bats coincide with periods of increased risk of human infection. PLoS Pathog..

[13] Amman B.R., Nyakarahuka L., McElroy A.K., Dodd K.A., Sealy T.K., Schuh A.J., Shoemaker T.R., Balinandi S., Atimnedi P., Kaboyo W. (2014). *Marburgvirus* resurgence in Kitaka Mine bat population after extermination attempts, Uganda. Emerg. Infect. Dis..

[14] Paweska J.T., Jansen van Vuren P., Masumu J., Leman P.A., Grobbelaar A.A., Birkhead M., Clift S., Swanepoel R., Kemp A. (2012). Virological and serological findings in *Rousettus aegyptiacus* experimentally inoculated with Vero cells-adapted hogan strain of Marburg virus. PLoS ONE.

[15] Amman B.R., Jones M.E., Sealy T.K., Uebelhoer L.S., Schuh A.J., Bird B.H., Coleman-McCray J.D., Martin B.E., Nichol S.T., Towner J.S. (2015). Oral shedding of Marburg virus in experimentally infected Egyptian fruit bats (*Rousettus aegyptiacus*). J. Wildl. Dis..

[16] Jones M.E., Schuh A.J., Amman B.R., Sealy T.K., Zaki S.R., Nichol S.T., Towner J.S. (2015). Experimental inoculation of Egyptian Rousette bats (*Rousettus aegyptiacus*) with viruses of the *Ebolavirus* and *Marburgvirus* genera. Viruses.

[17] Paweska J.T., Jansen van Vuren P., Fenton K.A., Graves K., Grobbelaar A.A., Moolla N., Leman P., Weyer J., Storm N., McCulloch S.D. (2015). Lack of Marburg virus transmission from experimentally infected to susceptible in-contact Egyptian fruit bats. J. Infect. Dis..

[18] Brauburger K., Deflubé L.R., Mühlberger E., Pattnaik A.K., Whitt M.A. (2015). Filovirus transcription and replication. Biology and Pathogenesis of Rhabdo- and Filoviruses.

[19] Feldmann H., Will C., Schikore M., Slenczka W., Klenk H.D. (1991). Glycosylation and oligomerization of the spike protein of Marburg virus. Virology.

[20] Hunt C.L., Lennemann N.J., Maury W. (2012). Filovirus entry: A novelty in the viral fusion world. Viruses.

[21] Miller E.H., Chandran K. (2012). Filovirus entry into cells—New insights. Curr. Opin. Virol..

[22] Volchkov V.E., Volchkova V.A., Stroher U., Becker S., Dolnik O., Cieplik M., Garten W., Klenk H.D., Feldmann H. (2000). Proteolytic processing of Marburg virus glycoprotein. Virology.

[23] Koellhoffer J.F., Malashkevich V.N., Harrison J.S., Toro R., Bhosle R.C., Chandran K., Almo S.C., Lai J.R. (2012). Crystal structure of the Marburg virus GP2 core domain in its postfusion conformation. Biochemistry.

[24] Fusco M.L., Hashiguchi T., Cassan R., Biggins J.E., Murin C.D., Warfield K.L., Li S., Holtsberg F.W., Shulenin S., Vu H. (2015). Protective mAbs and cross-reactive mAbs raised by immunization with engineered Marburg virus GPs. PLoS Pathog..

[25] Hashiguchi T., Fusco M.L., Bornholdt Z.A., Lee J.E., Flyak A.I., Matsuoka R., Kohda D., Yanagi Y., Hammel M., Crowe J.E. (2015). Structural basis for Marburg virus neutralization by a cross-reactive human antibody. Cell.

[26] Flyak A.I., Ilinykh P.A., Murin C.D., Garron T., Shen X., Fusco M.L., Hashiguchi T., Bornholdt Z.A., Slaughter J.C., Sapparapu G. (2015). Mechanism of human antibody-mediated neutralization of Marburg virus. Cell.

[27] Kibuuka H., Berkowitz N.M., Millard M., Enama M.E., Tindikahwa A., Sekiziyivu A.B., Costner P., Sitar S., Glover D., Hu Z. (2015). Safety and immunogenicity of Ebola virus and Marburg virus glycoprotein DNA vaccines assessed separately and concomitantly in healthy Ugandan adults: A phase 1b, randomised, double-blind, placebo-controlled clinical trial. Lancet.

[28] Daddario-DiCaprio K.M., Geisbert T.W., Stroher U., Geisbert J.B., Grolla A., Fritz E.A., Fernando L., Kagan E., Jahrling P.B., Hensley L.E. (2006). Postexposure protection against Marburg hemorrhagic fever with recombinant vesicular stomatitis virus vectors in non-human primates: An efficacy assessment. Lancet.

[29] Kolesnikova L., Bohil A.B., Cheney R.E., Becker S. (2007). Budding of *Marburgvirus* is associated with filopodia. Cell. Microbiol..

[30] Kolesnikova L., Mittler E., Schudt G., Shams-Eldin H., Becker S. (2012). Phosphorylation of Marburg virus matrix protein VP40 triggers assembly of nucleocapsids with the viral envelope at the plasma membrane. Cell. Microbiol..

[31] Wijesinghe K.J., Stahelin R.V. (2015). Investigation of the lipid binding properties of the Marburg virus matrix protein VP40. J. Virol..

[32] Valmas C., Basler C.F. (2011). Marburg virus VP40 antagonizes interferon signaling in a species-specific manner. J. Virol..

[33] Valmas C., Grosch M.N., Schümann M., Olejnik J., Martinez O., Best S.M., Krähling V., Basler C.F., Mühlberger E. (2010). Marburg virus evades interferon responses by a mechanism distinct from Ebola virus. PLoS Pathog..

[34] Feagins A.R., Basler C.F. (2015). Amino acid residue at position 79 of Marburg virus VP40 confers interferon antagonism in mouse cells. J. Infect. Dis..

[35] Feagins A.R., Basler C.F. (2014). The VP40 protein of Marburg virus exhibits impaired budding and increased sensitivity to human tetherin following mouse adaptation. J. Virol..

[36] Koehler A., Kolesnikova L., Welzel U., Schudt G., Herwig A., Becker S. (2015). A single amino acid change in the Marburg virus matrix protein VP40 provides a replicative advantage in a species-specific manner. J. Virol..

[37] Bharat T.A., Riches J.D., Kolesnikova L., Welsch S., Krahling V., Davey N., Parsy M.L., Becker S., Briggs J.A. (2011). Cryo-electron tomography of Marburg virus particles and their morphogenesis within infected cells. PLoS Biol..

[38] Becker S., Rinne C., Hofsäss U., Klenk H.-D., Mühlberger E. (1998). Interactions of Marburg virus nucleocapsid proteins. Virology.

[39] Bamberg S., Kolesnikova L., Möller P., Klenk H.D., Becker S. (2005). VP24 of Marburg virus influences formation of infectious particles. J. Virol..

[40] Wenigenrath J., Kolesnikova L., Hoenen T., Mittler E., Becker S. (2010). Establishment and application of an infectious virus-like particle system for Marburg virus. J. Gen. Virol..

[41] Page A., Volchkova V.A., Reid S.P., Mateo M., Bagnaud-Baule A., Nemirov K., Shurtleff A.C., Lawrence P., Reynard O., Ottmann M. (2014). *Marburgvirus* hijacks Nrf2-dependent pathway by targeting Nrf2-negative regulator Keap1. Cell Rep..

[42] Edwards M.R., Johnson B., Mire C.E., Xu W., Shabman R.S., Speller L.N., Leung D.W., Geisbert T.W., Amarasinghe G.K., Basler C.F. (2014). The Marburg virus VP24 protein interacts with Keap1 to activate the cytoprotective antioxidant response pathway. Cell Rep..

[43] Kolesnikova L., Mühlberger E., Ryabchikova E., Becker S. (2000). Ultrastructural organization of recombinant Marburg virus nucleoprotein: Comparison with Marburg virus inclusions. J. Virol..

[44] Mavrakis M., Kolesnikova L., Schoehn G., Becker S., Ruigrok R.W. (2002). Morphology of Marburg virus NP-RNA. Virology.

[45] Dolnik O., Kolesnikova L., Stevermann L., Becker S. (2010). Tsg101 is recruited by a late domain of the nucleocapsid protein to support budding of Marburg virus-like particles. J. Virol..

[46] Dolnik O., Kolesnikova L., Welsch S., Strecker T., Schudt G., Becker S. (2014). Interaction with Tsg101 is necessary for the efficient transport and release of nucleocapsids in marburg virus-infected cells. PLoS Pathog..

[47] Dolnik O., Stevermann L., Kolesnikova L., Becker S. (2015). Marburg virus inclusions: A virus-induced microcompartment and interface to multivesicular bodies and the late endosomal compartment. Eur. J. Cell Biol..

[48] Leung D.W., Borek D., Luthra P., Binning J.M., Anantpadma M., Liu G., Harvey I.B., Su Z., Endlich-Frazier A., Pan J. (2015). An intrinsically disordered peptide from Ebola virus VP35 controls viral RNA synthesis by modulating nucleoprotein-RNA interactions. Cell Rep..

[49] Kirchdoerfer R.N., Abelson D.M., Li S., Wood M.R., Saphire E.O. (2015). Assembly of the Ebola virus nucleoprotein from a chaperoned VP35 complex. Cell Rep..

[50] Möller P., Pariente N., Klenk H.D., Becker S. (2005). Homo-oligomerization of *Marburgvirus* VP35 is essential for its function in replication and transcription. J. Virol..

[51] Mühlberger E. (2007). Filovirus replication and transcription. Future Virol..

[52] Bale S., Julien J.P., Bornholdt Z.A., Kimberlin C.R., Halfmann P., Zandonatti M.A., Kunert J., Kroon G.J., Kawaoka Y., MacRae I.J. (2012). Marburg virus VP35 can both fully coat the backbone and cap the ends of dsRNA for interferon antagonism. PLoS Pathog..

[53] Ramanan P., Edwards M.R., Shabman R.S., Leung D.W., Endlich-Frazier A.C., Borek D.M., Otwinowski Z., Liu G., Huh J., Basler C.F. (2012). Structural basis for Marburg virus VP35-mediated immune evasion mechanisms. Proc. Natl. Acad. Sci. USA.

[54] Albarino C.G., Wiggleton Guerrero L., Spengler J.R., Uebelhoer L.S., Chakrabarti A.K., Nichol S.T., Towner J.S. (2015). Recombinant Marburg viruses containing mutations in the IID region of VP35 prevent inhibition of host immune responses. Virology.

[55] Edwards M.R., Liu G., Mire C.E., Sureshchandra S., Luthra P., Yen B., Shabman R.S., Leung D.W., Messaoudi I., Geisbert T.W. (2016). Differential regulation of interferon responses by Ebola and Marburg virus VP35 proteins. Cell Rep..

[56] Yen B.C., Basler C.F. (2016). Effects of filovirus IFN antagonists on responses of human monocyte-derived dendritic cells to RNA virus infection. J. Virol..

[57] Modrof J., Moritz C., Kolesnikova L., Konakova T., Hartlieb B., Randolf A., Muhlberger E., Becker S. (2001). Phosphorylation of Marburg virus VP30 at serines 40 and 42 is critical for its interaction with NP inclusions. Virology.

[58] Mühlberger E., Weik M., Volchkov V.E., Klenk H.-D., Becker S. (1999). Comparison of the transcription and replication strategies of marburg virus and Ebola virus by using artificial replication systems. J. Virol..

[59] Weik M., Modrof J., Klenk H.D., Becker S., Mühlberger E. (2002). Ebola virus VP30-mediated transcription is regulated by RNA secondary structure formation. J. Virol..

[60] Mühlberger E., Lötfering B., Klenk H.-D., Becker S. (1998). Three of the four nucleocapsid proteins of Marburg virus, NP, VP35, and L, are sufficient to mediate replication and transcription of Marburg virus-specific monocistronic minigenomes. J. Virol..

[61] Albarino C.G., Uebelhoer L.S., Vincent J.P., Khristova M.L., Chakrabarti A.K., McElroy A., Nichol S.T., Towner J.S. (2013). Development of a reverse genetics system to generate recombinant Marburg virus derived from a bat isolate. Virology.

[62] Enterlein S., Volchkov V., Weik M., Kolesnikova L., Volchkova V., Klenk H.D., Mühlberger E. (2006). Rescue of recombinant Marburg virus from cDNA is dependent on nucleocapsid protein VP30. J. Virol..

[63] Fowler T., Bamberg S., Möller P., Klenk H.D., Meyer T.F., Becker S., Rudel T. (2005). Inhibition of Marburg virus protein expression and viral release by RNA interference. J. Gen. Virol..

[64] Feldmann H., Mühlberger E., Randolf A., Will C., Kiley M.P., Sanchez A., Klenk H.D. (1992). Marburg virus, a filovirus: Messenger RNAs, gene order, and regulatory elements of the replication cycle. Virus Res..

[65] Mühlberger E., Trommer S., Funke C., Volchkov V., Klenk H.-D., Becker S. (1996). Termini of all mRNA species of Marburg virus: Sequence and secondary structure. Virology.

[66] Enterlein S., Schmidt K.M., Schümann M., Conrad D., Krahling V., Olejnik J., Mühlberger E. (2009). The Marburg virus 3' non-coding region structurally and functionally differs from that of Ebola virus. J. Virol..

[67] Whelan S.P., Barr J.N., Wertz G.W. (2004). Transcription and replication of nonsegmented negative-strand RNA viruses. Curr. Top. Microbiol. Immunol..

[68] Hofmann-Winkler H., Kaup F., Pohlmann S. (2012). Host cell factors in filovirus entry: Novel players, new insights. Viruses.

[69] Saeed M.F., Kolokoltsov A.A., Albrecht T., Davey R.A. (2010). Cellular entry of Ebola virus involves uptake by a macropinocytosis-like mechanism and subsequent trafficking through early and late endosomes. PLoS Pathog..

[70] Nanbo A., Imai M., Watanabe S., Noda T., Takahashi K., Neumann G., Halfmann P., Kawaoka Y. (2010). *Ebolavirus* is internalized into host cells via macropinocytosis in a viral glycoprotein-dependent manner. PLoS Pathog..

[71] Misasi J., Chandran K., Yang J.Y., Considine B., Filone C.M., Cote M., Sullivan N., Fabozzi G., Hensley L., Cunningham J. (2012). Filoviruses require endosomal cysteine proteases for entry but exhibit distinct protease preferences. J. Virol..

[72] Gnirss K., Kuhl A., Karsten C., Glowacka I., Bertram S., Kaup F., Hofmann H., Pohlmann S. (2012). Cathepsins B and L activate Ebola but not Marburg virus glycoproteins for efficient entry into cell lines and macrophages independent of TMPRSS2 expression. Virology.

[73] Carette J.E., Raaben M., Wong A.C., Herbert A.S., Obernosterer G., Mulherkar N., Kuehne A.I., Kranzusch P.J., Griffin A.M., Ruthel G. (2011). Ebola virus entry requires the cholesterol transporter niemann-pick C1. Nature.

[74] Cote M., Misasi J., Ren T., Bruchez A., Lee K., Filone C.M., Hensley L., Li Q., Ory D., Chandran K. (2011). Small molecule inhibitors reveal Niemann-Pick C1 is essential for Ebola virus infection. Nature.

[75] Liu N., Tao Y., Brenowitz M.D., Girvin M.E., Lai J.R. (2015). Structural and functional studies on the Marburg virus GP2 fusion loop. J. Infect. Dis..

[76] Ryabchikova E., Price B.B.S. (2004). Ebola and Marburg Viruses: A View of Infection Using Electron Microscopy.

[77] Schmidt K.M., Schümann M., Olejnik J., Krähling V., Mühlberger E. (2011). Recombinant Marburg virus expressing EGFP allows rapid screening of virus growth and real-time visualization of virus spread. J. Infect. Dis..

[78] Kolesnikova L., Berghofer B., Bamberg S., Becker S. (2004). Multivesicular bodies as a platform for formation of the Marburg virus envelope. J. Virol..

[79] Welsch S., Kolesnikova L., Krahling V., Riches J.D., Becker S., Briggs J.A. (2010). Electron tomography reveals the steps in filovirus budding. PLoS Pathog..

[80] Schudt G., Kolesnikova L., Dolnik O., Sodeik B., Becker S. (2013). Live-cell imaging of Marburg virus-infected cells uncovers actin-dependent transport of nucleocapsids over long distances. Proc. Natl. Acad. Sci. USA.

[81] Mittler E., Kolesnikova L., Strecker T., Garten W., Becker S. (2007). Role of the transmembrane domain of marburg virus surface protein GP in assembly of the viral envelope. J. Virol..

[82] Sänger C., Mühlberger E., Ryabchikova E., Kolesnikova L., Klenk H.D., Becker S. (2001). Sorting of Marburg virus surface protein and virus release take place at opposite surfaces of infected polarized epithelial cells. J. Virol..

[83] Kolesnikova L., Ryabchikova E., Shestopalov A., Becker S. (2007). Basolateral budding of Marburg virus: VP40 retargets viral glycoprotein GP to the basolateral surface. J. Infect. Dis..

[84] Schnittler H.J., Mahner F., Drenckhahn D., Klenk H.D., Feldmann H. (1993). Replication of Marburg virus in human endothelial cells. A possible mechanism for the development of viral hemorrhagic disease. J. Clin. Invest..

[85] Conzelmann K.K. (2004). Reverse genetics of mononegavirales. Curr. Top. Microbiol. Immunol..

[86] Hoenen T., Groseth A., de Kok-Mercado F., Kuhn J.H., Wahl-Jensen V. (2011). Minigenomes, transcription and replication competent virus-like particles and beyond: Reverse genetics systems for filoviruses and other negative stranded hemorrhagic fever viruses. Antivir. Res..

[87] Neumann G., Kawaoka Y. (2004). Reverse genetics systems for the generation of segmented negative-sense RNA viruses entirely from cloned cDNA. Curr. Top. Microbiol. Immunol..

[88] Lötfering B., Mühlberger E., Tamura T., Klenk H.D., Becker S. (1999). The nucleoprotein of Marburg virus is target for multiple cellular kinases. Virology.

[89] DiCarlo A., Moller P., Lander A., Kolesnikova L., Becker S. (2007). Nucleocapsid formation and RNA synthesis of Marburg virus is dependent on two coiled coil motifs in the nucleoprotein. Virol. J..

[90] Alonso J.A., Patterson J.L. (2013). Sequence variability in viral genome non-coding regions likely contribute to observed differences in viral replication amongst MARV strains. Virology.

[91] Uebelhoer L.S., Albarino C.G., McMullan L.K., Chakrabarti A.K., Vincent J.P., Nichol S.T., Towner J.S. (2014). High-throughput, luciferase-based reverse genetics systems for identifying inhibitors of Marburg and Ebola viruses. Antivir. Res..

[92] DiCarlo A., Biedenkopf N., Hartlieb B., Klussmeier A., Becker S. (2011). Phosphorylation of Marburg virus NP region II modulates viral RNA synthesis. J. Infect. Dis..

[93] Mittler E., Kolesnikova L., Hartlieb B., Davey R., Becker S. (2011). The cytoplasmic domain of Marburg virus GP modulates early steps of viral infection. J. Virol..

[94] Spiegelberg L., Wahl-Jensen V., Kolesnikova L., Feldmann H., Becker S., Hoenen T. (2011). Genus-specific recruitment of filovirus ribonucleoprotein complexes into budding particles. J. Gen. Virol..

[95] Krähling V., Dolnik O., Kolesnikova L., Schmidt-Chanasit J., Jordan I., Sandig V., Gunther S., Becker S. (2010). Establishment of fruit bat cells (*Rousettus aegyptiacus*) as a model system for the investigation of filoviral infection. PLoS Negl. Trop. Dis..

[96] Mittler E., Kolesnikova L., Herwig A., Dolnik O., Becker S. (2013). Assembly of the Marburg virus envelope. Cell. Microbiol..

[97] Theriault S., Groseth A., Neumann G., Kawaoka Y., Feldmann H. (2004). Rescue of Ebola virus from cDNA using heterologous support proteins. Virus Res..

[98] Pattnaik A.K., Ball L.A., LeGrone A.W., Wertz G.W. (1992). Infectious defective interfering particles of VSV from transcripts of a cDNA clone. Cell.

[99] Pattnaik A.K., Wertz G.W. (1990). Replication and amplification of defective interfering particle RNAs of vesicular stomatitis virus in cells expressing viral proteins from vectors containing cloned cDNAs. J. Virol..

[100] Ball L.A. (1992). Cellular expression of a functional nodavirus RNA replicon from vaccinia virus vectors. J. Virol..

[101] Neumann G., Feldmann H., Watanabe S., Lukashevich I., Kawaoka Y. (2002). Reverse genetics demonstrates that proteolytic processing of the Ebola virus glycoprotein is not essential for replication in cell culture. J. Virol..

[102] Buchholz U.J., Finke S., Conzelmann K.K. (1999). Generation of bovine respiratory syncytial virus (BRSV) from cDNA: BRSV NS2 is not essential for virus replication in tissue culture, and the human RSV leader region acts as a functional BRSV genome promoter. J. Virol..

[103] Sutter G., Ohlmann M., Erfle V. (1995). Non-replicating vaccinia vector efficiently expresses bacteriophage T7 RNA polymerase. FEBS Lett..

[104] Bali V., Bebok Z. (2015). Decoding mechanisms by which silent codon changes influence protein biogenesis and function. Int. J. Biochem. Cell Biol..

[105] Swenson D.L., Warfield K.L., Kuehl K., Larsen T., Hevey M.C., Schmaljohn A., Bavari S., Aman M.J. (2004). Generation of Marburg virus-like particles by co-expression of glycoprotein and matrix protein. FEMS Immunol. Med. Microbiol..

[106] Kolesnikova L., Bamberg S., Berghofer B., Becker S. (2004). The matrix protein of Marburg virus is transported to the plasma membrane along cellular membranes: Exploiting the retrograde late endosomal pathway. J. Virol..

[107] Schnell M.J., Mebatsion T., Conzelmann K.K. (1994). Infectious rabies viruses from cloned cDNA. EMBO J..

[108] Kato A., Sakai Y., Shioda T., Kondo T., Nakanishi M., Nagai Y. (1996). Initiation of Sendai virus multiplication from transfected cDNA or RNA with negative or positive sense. Genes Cells.

[109] Durbin A.P., Hall S.L., Siew J.W., Whitehead S.S., Collins P.L., Murphy B.R. (1997). Recovery of infectious human parainfluenza virus type 3 from cDNA. Virology.

[110] Pfaller C.K., Cattaneo R., Schnell M.J. (2015). Reverse genetics of Mononegavirales: How they work, new vaccines, and new cancer therapeutics. Virology.

[111] Falzarano D., Groseth A., Hoenen T. (2014). Development and application of reporter-expressing mononegaviruses: Current challenges and perspectives. Antivir. Res..

